# Butein Increases Resistance to Oxidative Stress and Lifespan with Positive Effects on the Risk of Age-Related Diseases in *Caenorhabditis elegans*

**DOI:** 10.3390/antiox13020155

**Published:** 2024-01-26

**Authors:** Seona Kim, Hyemin Yoon, Sang-Kyu Park

**Affiliations:** 1 Department of Medical Sciences, General Graduate School, Soonchunhyang University, 22 Soonchunhyang-ro, Asan 31538, Republic of Korea; 2 Department of Medical Biotechnology, Soonchunhyang University, 22 Soonchunhyang-ro, Asan 31538, Republic of Korea

**Keywords:** butein, oxidative stress, lifespan, age-related diseases, *C. elegans*

## Abstract

Butein is a flavonoid found in many plants, including dahlia, butea, and coreopsis, and has both antioxidant and sirtuin-activating activities. In light of the postulated role of free radicals in aging, we examined the effects of butein on aging and on genetic or nutritional models of age-related diseases in *Caenorhabditis elegans*. Butein showed radical scavenging activity and increased resistance to oxidative stress in *Caenorhabditis elegans*. The mean lifespan of *Caenorhabditis elegans* was significantly increased by butein, from 22.7 days in the untreated control to 25.0 days in the butein-treated group. However, the lifespan-extending effect of butein was accompanied by reduced production of progeny as a trade-off. Moreover, the age-related decline in motility was delayed by butein supplementation. Genetic analysis showed that the lifespan-extending effect of butein required the autophagic protein BEC-1 and the transcription factor DAF-16 to regulate stress response and aging. At the genetic level, expression of the DAF-16 downstream target genes *hsp-16.2* and *sod-3* was induced in butein-treated worms. Butein additionally exhibited a preventive effect in models of age-related diseases. In an Alzheimer’s disease model, butein treatment significantly delayed the paralysis caused by accumulation of amyloid-beta in muscle, which requires SKN-1, not DAF-16. In a high-glucose-diet model of diabetes mellitus, butein markedly improved survival, requiring both SKN-1 and DAF-16. In a Parkinson’s disease model, dopaminergic neurodegeneration was completely inhibited by butein supplementation and the accumulation of α-synuclein was significantly reduced. These findings suggest the use of butein as a novel nutraceutical compound for aging and age-related diseases.

## 1. Introduction

Reactive oxygen species (ROS), including hydroxyl radical (HO·), hydroxide ion (HO^−^), superoxide anion (O2·^−^), and hydrogen peroxide (H_2_O_2_), are formed by the reduction of molecular oxygen. They are byproducts of mitochondrial oxidative phosphorylation, but are also formed by intracellular organelles such as peroxisomes and chloroplasts [1]. Pollutants, heavy metals, and ionizing radiation can lead to ROS formation in cells as well. Under normal conditions, ROS are present in cells at low levels and are required for cellular signaling pathways, homeostasis, and the response to cellular stress; however, in excessive amounts, they can cause oxidative damage to DNA, proteins, lipids, and other cellular macromolecules [2]. According to the free radical theory of aging, the age-related accumulation of oxidative damage by ROS is among the mechanisms of aging [3]. Age-related ROS accumulation increases both apoptosis in cardiomyocyte, a major hallmark of the aged heart, and neuroinflammation in the aged brain, which can result in neurodegeneration [4]. ROS have also been implicated in the development of many age-related diseases, including cancers, through the expansion of cancer cells via MAPK and PI3K signaling [5]. Cell migration and invasion are activated by ROS, as is angiogenesis, which is necessary to support metastasis [6]. Increased oxidative damage to DNA, proteins, and lipids has been reported in patients with Alzheimer’s disease (AD), Parkinson’s disease (PD), and amyotrophic lateral sclerosis [7,8]. In both AD and PD, supplementation with antioxidants has been shown to inhibit the accumulation of the amyloid beta (Aβ) and α-synuclein proteins, which are respectively associated with the development of these diseases [9,10,11].

Antioxidant molecules such as vitamins, polyphenols, and flavonoids mainly work as ROS scavengers. The effects of antioxidant compounds provided as a dietary intervention on aging have been examined in various model organisms. Among these organisms, *C. elegans* has been widely employed as an aging model due to its conserved aging signaling with mammals, short life cycle, and applicable genetic manipulations. Over the past decade, many antioxidant molecules with anti-aging and lifespan-extending properties have been identified in *C. elegans* [12]. Plant-derived phytochemicals, including polyphenols, terpenoids, and alkaloids, are the most observed and promising anti-aging molecules discovered from *C. elegans* screening thus far [12]. Resveratrol, a polyphenol found in red wine, has shown the ability to retard many age-related pathophysiological changes and extend the lifespan of *C. elegans* [13]. Dietary supplementation with myricetin, a polyphenolic compound found abundantly in navel oranges and blueberry leaves, increased the lifespan of *C. elegans* [14]. Silymarin, a flavanol derivative, inhibited Aβ-induced toxicity and had lifespan-extending effects [15]. Anti-aging effects and preventive effects on age-related diseases, including AD, PD, and diabetes mellitus (DM), have been described following dietary supplementation with phlorizin, a flavonoid found in *Malus genus*, through a mechanism involving the DAF-16-induced stress response and autophagy [10]. Fisetin (3,3′,4′,7-tetrahydroxyflavone) reduced cellular ROS levels and increased both the mean and maximum lifespan of *C. elegans* [9].

Butein (3,4,2′,4′-tetrahydroxychalcone) is a flavonoid found in extracts from many plants; its pharmacological properties include antioxidant, anti-inflammatory, and antimicrobial activities [16,17]. Butein has shown the ability to reduce ethanol-induced ROS production in hepatic stellate cells and nitric oxide production in pancreatic β cells [18]. Neuroprotective effects of butein due to decreased levels of ROS and apoptotic cell death have been reported, as have inhibition of both lipogenesis and the expression of adipogenic markers in animals [19,20]. A recent study described butein-mediated activation of autophagy via AMPK/mTOR signaling in human chondrocytes [21]. The potential role of butein as a therapeutic agent in cancers such as neuroblastoma and breast cancer has been examined as well [22,23]. Dietary supplementation with butein led to recovery in an animal model of nonalcoholic fatty liver disease via reduced levels of ROS and inflammatory cytokines [24]. In a model of chronic heart failure, butein ameliorated cardiac injury and dysfunction through decreased ROS production [25].

In the present study, we examined the antioxidant and anti-aging effects of butein in *C. elegans*. The cellular mechanisms involved in butein-mediated lifespan extension were investigated in genetic analyses using long-lived genetic mutants, genetic knockdown, and target gene expression. The effects of dietary supplementation with butein on age-related diseases were investigated using genetic and nutritional disease models.

## 2. Materials and Methods

### 2.1. Worm Strains and Maintenance

All strains used for this study were purchased from the *C. elegans* Genetics Center (Minneapolis, MN, USA). The N2 strain was used as the wild-type strain. CL2070 (dvIs70 [*Phsp-6.2::GFP*, *rol-6*]) and CF1553 (muIs84 [*Psod-3::GFP*, *rol-6*]) express green fluorescence protein (GFP) under the *hsp-16.2* and *sod-3* promoters, respectively. For the identification of lifespan-extending mechanisms, three long-lived mutants were used: *age-1* (*hx546*), *clk-1* (*e2519*), and *eat-2* (*ad465*). The subcellular localization of DAF-16 was determined in TJ356 (zls356 IV [*daf-16p::daf-16a/b::GFP*, *rol-6*]). CL4176 (dvls27 [*myo-3/Aβ1-42/let UTR*, *rol-6*]) is a genetic model for AD in which human Aβ is expressed in *C. elegans* muscle tissue after induction. BZ555 (egIs1 [*dat-1p::GFP*], expressing GFP in dopaminergic neurons) and NL5901 (pkIs2386 [*unc-54p::alphasynuclein::YFP* + *unc-119(+)*], expressing yellow fluorescent α-synuclein in muscle) were used as genetic models for PD. The worms were maintained at 20 °C on nematode growth medium (NGM) plates (25 mM NaCl, 2.5 mg peptone/mL, 50 mM KPO_4_, 5 μg cholesterol/mL, 1 mM CaCl_2_, 1 mM MgSO_4_, and 1.7% agar) spread with *Escherichia coli* OP50 as the food source. Butein (Sigma Aldrich, Cat. No. 72795, St. Louis, MO, USA) was dissolved in 99.9% ethanol.

### 2.2. In Vitro Antioxidant Activity

Butein solution was mixed with the same volume of a freshly prepared ethanolic solution of 0.2 mM DPPH (2,2-diphenyl-1-picrylhydrazyl) in a 96-well plate. After incubation in the dark for 30 min at 37 °C, the absorbance at 517 nm (A) was measured with a spectrophotometer. The scavenging activity was calculated using the following equation: Inhibition of DPPH radical (%) = [A_control_ − (A_sample_ − A_blank_)/A_control_] × 100. Ascorbic acid (1 mg/mL) served as the positive control.

### 2.3. Resistance to Environmental Stressors

Age-synchronized 3-day-old adult worms (*n* = 30) were treated for 24 h with different concentrations of butein. To induce oxidative stress, the worms were transferred individually to S-basal medium without cholesterol (5.85 g sodium chloride, 1 g potassium phosphate dibasic, and 6 g potassium phosphate monobasic in 1 L sterilized distilled-water) containing 2 mM hydrogen peroxide (H_2_O_2_) in a 96-well plate. After 8 h of incubation, worm survival was examined under a microscope. Ultraviolet (UV) and heat stress were induced in age-synchronized 3-day-old worms (*n* = 60) by applying 1 min of UV irradiation (20 J/cm^2^/min) and 8 h of incubation at 35 °C, respectively. Live and dead worms were counted daily until all worms were dead.

### 2.4. Lifespan

Age-synchronized worms (*n* = 60) were grown on NGM plates containing 5-fluoro-2′-deoxyruridine (12.5 mg/L) to inhibit internal hatching (bagging). The number of live, dead, lost, killed, and bagged worms was recorded daily. Worms that were lost, killed, or bagged during the assay were classified as ‘censored’ and excluded from data analysis. The data were statistically analyzed using the log-rank test [26].

### 2.5. Fertility

Age-synchronized 2-day-old worms (*n* = 12) were randomly selected and transferred to fresh NGM plates (1 worm/plate) for 24 h, during which time they laid eggs. They were then transferred again to fresh NGM plates. Eggs left on the NGM plates were incubated at 20 °C for an additional 48 h and adult progeny were counted under microscope. This cycle was continued until no progeny were produced by each worm.

### 2.6. Motility

For the qualitative analysis of motility, the locomotive activity of worms at different ages was classified according to three phases: phase 1, spontaneous locomotive activity in the absence of any stimulus; phase 2, locomotive activity only in the presence of a mechanical stimulus; and phase 3, motility limited to the head and only in response to a mechanical stimulus (*n* = 100). Motility was quantitatively analyzed by counting the number of thrashing movements of the worms in M9 buffer during 1 min (*n* = 20).

### 2.7. RNAi

The expression of *daf-16*, *skn-1*, or *bec-1* was specifically inhibited using RNAi clones obtained from the Ahringer RNAi library and the bacterial feeding method [27]. Double-stranded RNA synthesis during bacterial culture was induced by the addition of isopropyl-β-D-thio-galactoside (Sigma-Aldrich) to the culture medium. Cultured bacteria carrying each RNAi clone were spread on NGM plates as a food source. An empty vector clone served as the negative control.

### 2.8. Subcellular Localization of DAF-16

Seven days after hatching, TJ356 worms were mounted on glass slides containing 1 M sodium azide. The subcellular distribution of GFP was monitored by fluorescence microscopy.

### 2.9. Expression of Downstream Targets of DAF-16

CL2070 and CF1553 worms were grown on NGM plates. Seven days after hatching, randomly selected worms were transferred to a 96-well plate and their fluorescence intensity was measured with a fluorescence multi-reader (Infinite F200, Tecan, Grodig, Austria) (*n* = 20). GFP was also observed by fluorescence microscopy in worms mounted on glass slides coated with 2% agarose and 1 M sodium azide.

### 2.10. Aβ-Induced Paralysis

Eggs laid by CL4176 worms were incubated for 24 h at 15 °C. Sixty randomly selected worms were then transferred to a 25 °C incubator for 24 h to induce human Aβ gene expression in muscle. After induction, the development of paralysis in the worms was monitored every hour until all worms were paralyzed.

### 2.11. Toxicity Caused by High-Glucose Diet (HGD)

Glucose toxicity was induced by spreading 100 μL of 40 mM glucose onto NGM plates prior to the addition of OP50. A lifespan assay was performed with 60 age-synchronized worms as previously described.

### 2.12. Genetic Model of PD

BZ555 worms were transferred to NGM containing 50 mM 6-hydroxydopamine (6-OHDA) and 10 mM ascorbic acid to induce degeneration of dopaminergic neurons and gently mixed every 10 min for 1 h at 20 °C. After three washes with M9 buffer, the worms were transferred to fresh NGM plates spread with OP50 and 12.5 mg/L of 5-fluoro-2-deoxyruridine, then incubated at 20 °C for 72 h. A fluorescence microscope fitted with a 470/22 nm excitation filter and a 525/50 nm emission filter was used to visualize the degeneration of dopaminergic neurons in worms mounted on glass slides. L-3,4-dihydroxyphenylalanine (L-DOPA)-treated worms served as the positive control for inhibition of dopaminergic neuronal degeneration. Three-day-old NL5901 worms were bleached and their eggs were cultured in M9 buffer for 24 h at 20 °C. Hatched worms were transferred to fresh NGM plates and incubated for 72 h at 20 °C. Fluorescent intensity was determined using a 500/24 nm excitation filter and a 542/27 nm emission filter. The fluorescence intensity observed in the head region of both strains was quantified using Image-J software (version 1.54).

## 3. Results

### 3.1. Butein Scavenges Free Radicals and Increases Resistance to Oxidative Stress

**Figure 1:**
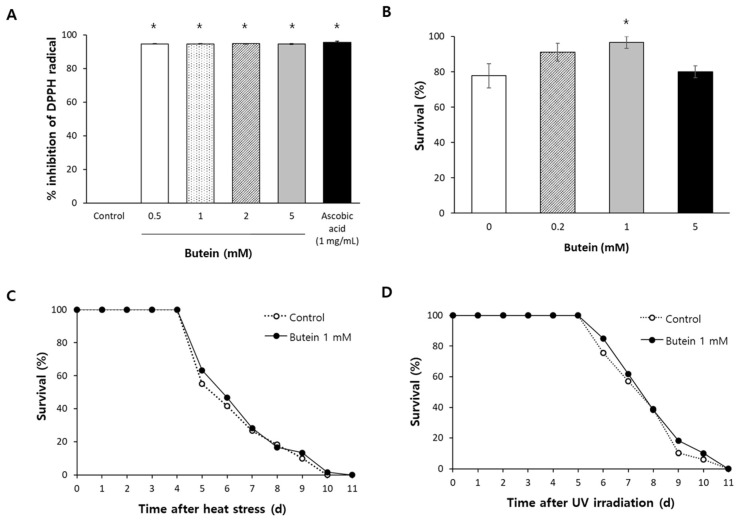
Effect of butein on the response to environmental stressors. (**A**) The ROS scavenging activity of different concentrations of butein was measured with DPPH radical in vitro. Ascorbic acid (1 mg/mL) served as the positive control. (**B**) Survival under oxidative stress conditions induced by H_2_O_2_ was examined in vivo. Age-synchronized young adults (*n* = 30) were used for each group. Survival of worms (*n* = 60) after heat shock (**C**) or UV irradiation (**D**) in the untreated control and the group treated with 1 mM butein was compared. Error bar indicates the standard error. *, *p* < 0.05.

The antioxidant activity of butein was determined by measuring the in vitro radical-scavenging activity and in vivo survival under oxidative stress conditions. The percent inhibitive effects of the well-known antioxidant ascorbic acid (1 mg/mL) was 95.6 ± 0.47% (mean ± standard error, *p* < 0.001 compared to the untreated control). All tested concentrations of butein showed strong radical-scavenging activity (Figure 1A). The percent inhibitive effects were 94.7 ± 0.07, 94.7 ± 0.06, 94.8 ± 0.07, and 94.7 ± 0.12 in worms treated with butein of 0.5, 1, 2, and 5 mM, respectively; all *p*-values were lower than 0.001 compared to the untreated control. In *C. elegans* with oxidative stress induced by H_2_O_2_, pretreatment with 1 mM butein increased the survival rate from 77.8 ± 4.01% in the untreated control to 96.7 ± 1.92% (*p* = 0.013) (Figure 1B). However, in worms treated with a higher concentration (5 mM) of butein, the survival difference was not significant compared to the untreated control (80.0 ± 1.92%, *p* = 0.643). Based on the results of the in vitro and in vivo antioxidant assays, 1 mM of butein was used in subsequent experiments. The effect of dietary supplementation with butein on the response of *C. elegans* to heat stress or UV irradiation was examined as well, but no significant effect was observed in either condition (Figure 1C,D).

### 3.2. Butein Extends the Lifespan of C. elegans but Reduces Fertility

**Figure 2:**
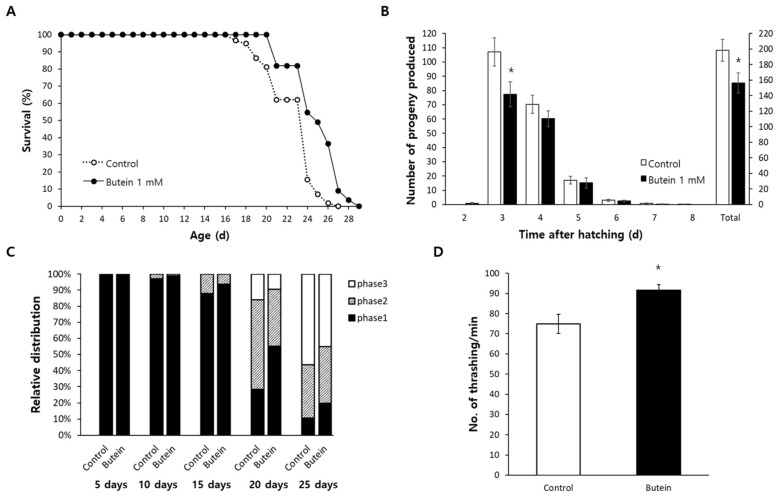
Anti-aging effect of butein supplementation. (**A**) Survival of worms was recorded daily until all worms were dead (with sixty age-synchronized worms). Butein significantly extended both the mean and the maximum lifespan. (**B**) The number of progeny produced by each worm (*n* = 12) was counted throughout a gravid period. Butein reduced the number of progeny produced during a gravid period. (**C**) Age-related changes in motility were analyzed qualitatively. Worms (*n* = 100) were classified according to their motility (phase 1, spontaneous movement in the absence of any stimulus; phase 2, motility only with in the presence of a mechanical stimulus; phase 3, movement only the head in response to a stimulus). (**D**) Quantitative analysis of motility was performed using a thrashing assay (*n* = 20). Age-related decline in motility was delayed by butein supplementation. The error bar indicates the standard error. *, *p* < 0.05.

According to the free radical theory of aging, cellular oxidative damage caused by free radicals is the major cause of aging [4]. In light of the increased resistance to oxidative stress observed in worms treated with butein, we next asked whether dietary supplementation with butein induced a longevity phenotype. The result of the lifespan assay showed that butein supplementation increased the mean lifespan from 22.7 days in the untreated control to 25.0 days in the butein-treated group (*p* < 0.001) and increased the maximum lifespan from 27 days to 29 days (Figure 2A). Independent replicative experiments showed a similar significant lifespan extension in response to butein (Table S1). However, many lifespan-extending genetic or nutritional interventions are accompanied by dysfunctional reproducibility as a trade-off [28,29]. This was the case in worms supplemented with butein, as the total number of progeny produced throughout a gravid period was significantly less than in the untreated control: 156.5 ± 12.88 vs. 198.4 ± 14.05 (*p* = 0.040). (Figure 2B). A comparison of the time-course distribution of progeny revealed a significant difference in the number of progeny produced on day 3: 107.1 ± 9.93 in the untreated control vs. 77.3 ± 8.84 in the butein-treated group (*p* = 0.036) (Figure 2B).

### 3.3. Butein Delays the Age-Related Decline in Motility

The effect of butein on the age-related decline in motility was examined both qualitatively and quantitatively. For the qualitative analysis, the worms were classified at different ages according to their locomotive activities. At young age (5 and 10 days after hatching), more than 97% of the worms showed active spontaneous locomotion in the absence of any mechanical stimulus (phase 1) in both the untreated control and the butein-treated group. Locomotive activity declined with aging, as the percentage of phase 1 worms decreased while the percentages of worms exhibiting locomotive activity only in the presence of a mechanical stimulus (phase 2) or movement only of the head in response to a stimulus (phase 3) increased with aging. However, butein supplementation delayed the age-related decline in motility, with an increase in the percentage of phase 1 for 20-day-old worms from 28.3% in the untreated control to 55.2% in the butein-treated group and a decrease in the percentage of worms of the same age in phase 3 from 16.2% to 9.4%, respectively. A similar effect was observed for 25-day-old worms (Figure 2C). A quantitative comparison of motility was performed through a thrashing assay. The number of thrashings per min was significantly higher in butein-treated worms (91.6 ± 2.86) than in the untreated control (74.8 ± 4.71; *p* = 0.007) (Figure 2D).

### 3.4. The Lifespan-Extending Effect of Butein Requires DAF-16 and BEC-1

**Figure 3:**
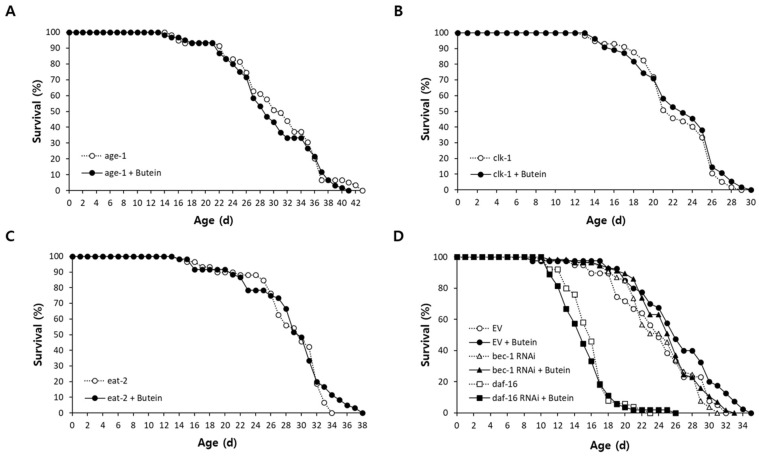
Underlying mechanisms involved in butein-induced longevity. Lifespans of the long-lived mutants *age-1* (**A**), *clk-1* (**B**), and *eat-2* (**C**) were compared in the untreated control and butein-treated group. There was no significant difference in lifespan with butein supplementation in any of the mutants. (**D**) Effect of genetic knockdown of *daf-16* or *bec-1* on the butein-induced lifespan extension examined using RNAi. The lifespan-extending effect of butein disappears when expression of *daf-16* or *bec-1* is inhibited. EV, empty vector.

The cellular mechanisms underlying butein-induced longevity were explored in three genetic mutants representing three lifespan-extending mechanisms. The mean lifespan of *age-1*, a mutant in which insulin/IGF-1-like signaling is reduced, was 30.6 days in the control and 30.0 days in the butein-treated group; the difference was not significant (*p* = 0.430) (Figure 3A). There was no significant increase in the mean lifespan of *clk-1*, in which ROS production is reduced, between the untreated control (22.4 days) and the butein-treated group (22.7 days; *p* = 0.473) (Figure 3B). Similarly, the difference was not significant in *eat-2* mutants, a genetic model of dietary restriction (DR), at 28.6 days in the untreated control and 28.8 days in the butein-treated group (*p* = 0.440) (Figure 3C). These results suggest that the mechanism underlying the extended lifespan conferred by butein involves pathways common to all three longevity mutants. Butein had no effect on the lifespan of worms with repressed expression of *bec-1*, a major autophagic gene in *C. elegans*. The genetic knockdown of *daf-16*, a FOXO transcription factor that regulates both stress response and aging, completely abolished the lifespan-extending effect of butein. (Figure 3D and Table S2).

### 3.5. DAF-16-Regulated Stress-Responsive Genes Are Induced by Butein

**Figure 4:**
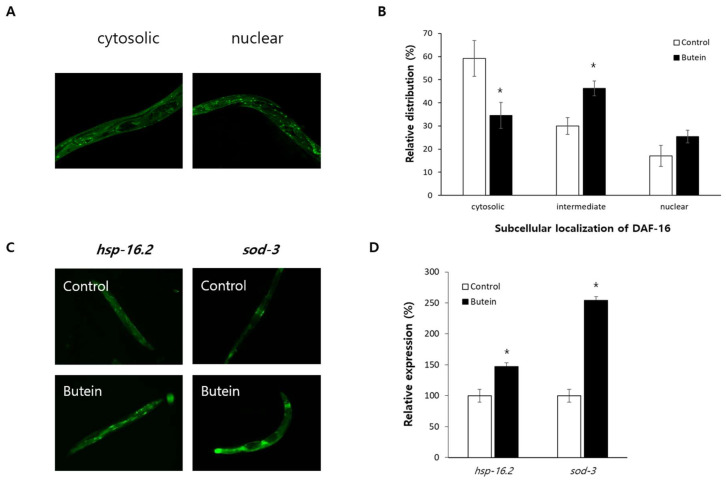
Effect of butein on the localization of DAF-16 and the expression of its target genes. The intracellular distribution of DAF-16 was monitored by confocal microscopy (**A**) and compared in the untreated control and the butein-treated group (**B**). (**C**) The effect of butein on the expression of GFP under the *hsp-16.2* or *sod-3* promoter was monitored by fluorescence microscopy. (**D**) The expression level of GFP in the untreated control and butein-treated group was quantitatively compared using a fluorescence multi-reader. The expression of *hsp-16.2* and *sod-3* was induced by butein supplementation. The error bar indicates the standard error. *, *p* < 0.05.

The effect of butein on the subcellular distribution of DAF-16 was examined using GFP fused to DAF-16. Dietary supplementation with butein enhanced the nuclear localization of DAF-16 (Figure 4A). The percentage of worms exhibiting a cytosolic distribution of GFP decreased from 59.2 ± 7.77% in the untreated control to 34.6 ± 5.58% in butein-treated worms (*p* = 0.042). The percentage of worms in which GFP was found in both the cytosol and the nucleus was higher in the butein-treated group than in the control, at 46.3 ± 3.22% vs. 30.0 ± 3.60% (*p* = 0.015). The percentage of worms in which GFP was present only in the nucleus increased from 17.1 ± 4.54% to 25.4 ± 2.76% by butein treatment (*p* = 0.168) (Figure 4B). The increased nuclear localization of DAF-16 led to upregulation of its downstream targets, as the expression of both *hsp-16.2* and *sod-3* was higher in butein-treated worms than in the untreated control: 146.9 ± 5.93% vs. 100.0 ± 10.33% (*p* = 0.001) and 254.0 ± 15.69% vs. 100.0 ± 12.83% (*p* < 0.001), respectively (Figure 4D).

### 3.6. Butein Prevents Aβ-Induced Toxicity in an AD Model

**Figure 5:**
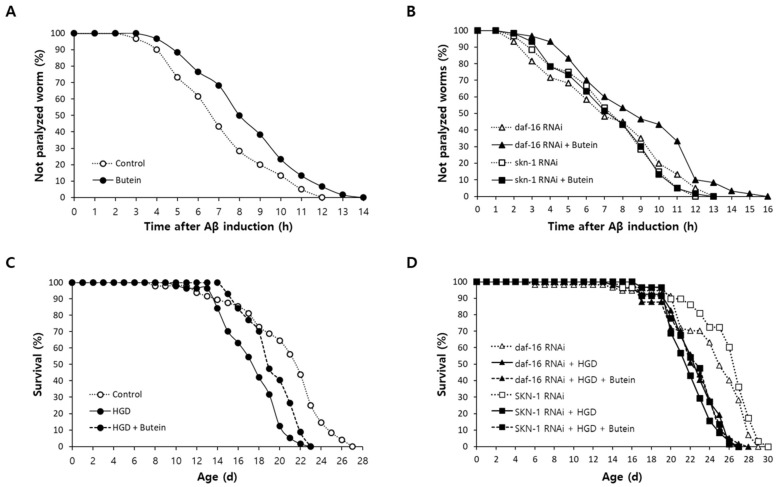
Effect of butein on Aβ and HGD toxicity. (**A**) The number of paralyzed worms after Aβ induction was recorded in the untreated control and butein-treated group until all worms were paralyzed. (**B**) The effect of gene knockdown on butein-induced protection against Aβ-induced paralysis was examined in worms fed *daf-16* or *skn-1* RNAi. Butein prevented Aβ-induced toxicity, requiring SKN-1 for its effect but not DAF-16. (**C**) Lifespans of the untreated control, HGD-treated, and HGD + butein-treated worms were compared in a lifespan assay. (**D**) The effect of butein on HGD-induced toxicity was monitored in worms fed *daf-16* or *skn-1* RNAi. Butein reduced HGD-induced motility, requiring both DAF-16 and SKN-1. HGD, high-glucose diet.

In the AD model of *C. elegans*, the muscle-specific expression of human Aβ causes paralysis. However, dietary supplementation with butein significantly inhibited Aβ-induced toxicity. Following Aβ induction, 50% of the untreated worms were paralyzed after 7.32 h and 100% were paralyzed after 12 h. In the butein-treated group, 50% of the worms were paralyzed at 8.63 h after Aβ induction (*p* = 0.016, 18.0% increase) and 100% were paralyzed at 14 h (Figure 5A). Genetic knockdown of *skn-1* but not *daf-16* abolished the inhibitory effect of butein on Aβ-induced toxicity. The inhibitory effect of butein on paralysis was similar in worms fed the *daf-16* RNAi clone, with a 21.8% delay in the time until 50% of the worms were paralyzed, from 7.4 h to 9.0 h (*p* = 0.018). However, when the expression of *skn-1* was inhibited, the time taken until 50% of the worms were paralyzed was not altered by butein treatment (*p* = 0.956) (Figure 5B). These findings suggest that the inhibitory effect of butein on Aβ-induced toxicity is dependent specifically on SKN-1, and not on DAF-16.

### 3.7. Butein Reduces HGD-Induced Mortality in C. elegans

HGD is a widely-used nutritional model for DM in *C. elegans* [30]. The mean lifespan of worms was reduced from 20.8 days in the untreated control to 17.5 days in HGD worms (*p* < 0.001). Following butein supplementation, the mean lifespan increased to 19.5 days (*p* = 0.002 vs. HGD worms) (Figure 5C). In addition, HGD significantly decreased the mean lifespan in worms treated with *daf-16* RNAi clone, from 24.5 days to 22.6 days (*p* < 0.001), although there was no improvement following butein supplementation, as the mean lifespan was essentially unchanged (22.5 days; *p* = 0.533). The increased mortality in HGD worms fed the *skn-1* clone was not ameliorated by butein. The mean lifespan of *skin-1* RNAi only, *skn-1* RNAi + HGD, and *skn-1* RNAi + HGD + butein worms was 25.8, 22.2, and 22.6 days, respectively. The difference between *skn-1* RNAi only vs. *skn-1* RNAi + HGD was significant (*p* < 0.001), while the difference between *skn-1* RNAi + HGD vs. *skn-1* RNAi + HGD + butein was not (*p* = 0.307) (Figure 5D).

### 3.8. Butein Inhibits Dopaminergic Neurodegeneration and Accumulation of α-Synuclein

**Figure 6:**
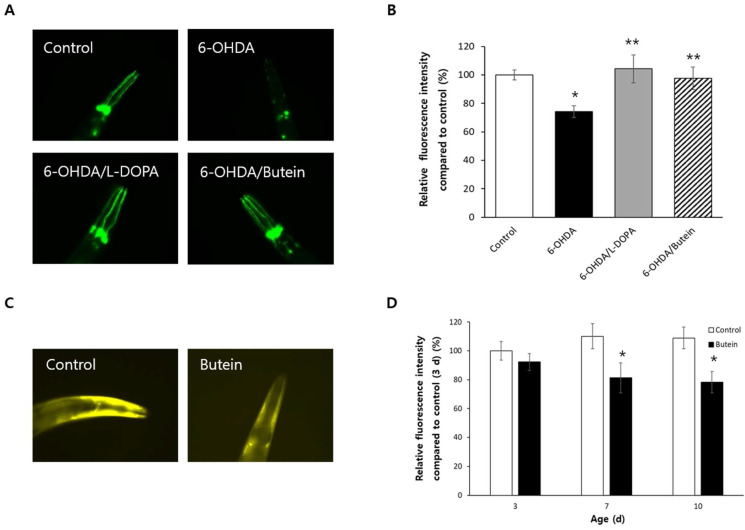
Neuroprotective potential of butein in a genetic model of PD. (**A**) Representative images of the untreated control and experimental BZ555 worms. (**B**) The fluorescence intensities of dopaminergic neurons were analyzed quantitatively using Image-J software. Butein prevented degeneration of dopaminergic neuron. The accumulation of α-synuclein was observed under fluorescence microscopy (**C**) and compared in the untreated control and butein-treated group at 3, 7, and 10 days after hatching (**D**). The accumulation of α-synuclein was inhibited by butein supplementation. The error bar indicates the standard error. *, *p* < 0.05 vs. control; **, *p* < 0.05 vs. 6-OHDA-treated group.

In strain BZ555 worms, both L-DOPA and butein prevented the 6-OHDA-induced neurodegeneration of GFP-expressing dopaminergic neurons (Figure 6A). The relative fluorescence intensity decreased from 100.0 ± 3.47% in the untreated control to 74.3 ± 4.12% in worms treated with 6-OHDA (*p* < 0.001), whereas the relative fluorescence intensity in the L-DOPA-treated and butein-treated groups was 104.3 ± 9.78 and 97.7 ± 7.88%, respectively (*p* < 0.05 vs. worms treated only with 6-OHDA) (Figure 6B). The effect of butein on the accumulation of α-synuclein was examined using strain NL5901 worms, which express fluorescent α-synuclein. The accumulation of α-synuclein decreased significantly in older worms treated with butein, whereas in younger worms there was no significant difference in fluorescence between the untreated control and the butein-treated group (Figure 6C). In butein-treated 7-day-old worms, the relative fluorescence intensity was reduced to 81.2 ± 10.50% vs. 110.1 ± 8.71% in the untreated control (*p* = 0.040). In 10-day-old worms, the relative fluorescence intensity was 108.9 ± 7.45% in the untreated control and 78.2 ± 7.47% in the butein-treated group (*p* = 0.007) (Figure 6D).

## 4. Discussion

This study examined the role of dietary-supplemented butein on the response to environmental stressors and aging using *C. elegans* as a model system. Among the tested environmental stressors, butein conferred resistance to oxidative stress but not to heat stress or UV irradiation. Both mean and maximum lifespan were significantly extended by supplementation with butein. These findings indicate that the impact of butein on longevity is mediated by its antioxidant activity, providing support for the free radical theory of aging. Previous studies have reported that quercetin and fisetin, both of which are phenolic flavonols, can increase the lifespan of yeast and nematodes [9,31]. Dietary supplementation with the flavonoid kaempferol has been shown to extend the lifespan of *C. elegans*, with its effect being mediated by DAF-16 [32]. According to the disposable soma theory, organisms allocate their limited cellular resources between growth, reproduction, and age-related maintenance, which would explain why many genetic or nutritional prolongations of an organism’s lifespan are accompanied by a functional trade-off [33]. For example, organisms fed resveratrol or fisetin have a longer lifespan but reduced fertility [9,28]. Similarly, a long-lived mutant, *age-1*, produces fewer progeny than the wild-type control [29]. The longevity phenotype observed with butein supplementation was accompanied by fewer progeny, implying re-allocation of cellular resources from reproduction to maintenance and repair. An age-related decline in motility is a strong biomarker of aging, as it reflects universal and progressive features of the aging process. In this study, qualitative and quantitative analyses showed that butein delayed the age-related decline in motility. This delay in muscle aging may be due to the antioxidant activities of butein, as age-related changes in muscle, including atrophy, sarcopenia, and increased apoptosis, are consequences of increased ROS production in aged muscles [34].

The mechanisms underlying the butein-induced prolongation of the lifespan of *C. elegans* were investigated in long-lived mutants. Butein supplementation did not further extend the lifespan of the genetic mutants *age-1*, *clk-1*, and *eat-2*, indicating that its impact on longevity is conferred by a mechanism common to all three lifespan-regulating pathways. Recent studies have reported that autophagy is required for the prolonged lifespan mediated by reduced insulin/IGF-1-like signaling, DR, or decreased mitochondrial respiration [35]. Genetic knockout of the autophagy genes *bec-1* and *unc-51* suppressed the prolonged lifespans of the *daf-2* and *eat-2* genetic models of reduced insulin/IGF-1-like signaling and DR in *C. elegans*, respectively [35]. In *C. elegans* with *clk-1* knockdown, the longevity phenotype of the *bec-1* and *unc-51* mutants was eliminated [35]. DAF-16 is a FOXO-transcription factor that modulates the response to oxidative stress and is required for the activity of lifespan-extending pathways, including the reduced insulin/IGF-1-like signaling and DR pathways [36]. Dietary supplementation with butein induced nuclear localization of DAF-16 and increased the expression of its downstream targets *hsp-16.2* and *sod-3*. However, RNAi-mediated repression of either *bec-1* or *daf-16* completely inhibited the butein-mediated lifespan extension. These results suggest that the lifespan-prolonging effects of butein require autophagy and the DAF-16-induced stress response. Previous studies of *C. elegans* with the same genetic background showed a positive correlation of lifespan with the expression levels of *hsp-16.2* and *sod-3* [37,38]. In young worms exposed to heat stress, *hsp-16.2* expression correlated positively with individual’s thermotolerance and life expectancy. Worms with higher levels of *hsp-16.2* expression lived longer than worms with lower levels of *hsp-16.2* expression, suggesting that *hsp-16.2* expression level is a major predictor of the remaining lifespan of *C. elegans* [37]. Genetic screening in *C. elegans* for genes responsible for aging variability and regulating lifespan identified *sod-3* as the best single longevity-promoting gene, as its increased expression resulted in a 22% longer lifespan than that in worms expressing less *sod-3* [38]. Therefore, these results indicate that the butein-induced prolongation of lifespan is mediated the induction of longevity-promoting genes by DAF-16.

ROS have been implicated in many age-related diseases, including cancer, neurodegenerative diseases, and DM, while recent studies have demonstrated preventive or therapeutic effects on these diseases of dietary supplementation with antioxidant flavonoids [1,8]. Quercetin, a flavonoid found in red wine, has been shown to prevent the formation of Aβ fibrils, while the flavanone derivative silymarin delayed the paralysis caused by Aβ accumulation in AD animal models [15,39]. Fisetin, a plant-derived flavonoid, suppressed Aβ plaque formation and tau-mediated neurofibrillary tangles [40,41]. In another study, it reduced HGD-induced toxicity in a disease model of DM and blocked the degeneration of dopaminergic neurons in a PD model [9]. Dietary supplementation with phlorizin conferred protective effects on Aβ- and HGD-induced toxicities and inhibited 6-OHDA-induced neurodegeneration [10]. In the present study, butein decreased Aβ-induced paralysis through a pathway involving DAF-16 but not SKN-1. In other studies, the prevention of Aβ-induced toxicity for otophylloside B was likewise dependent on DAF-16 but not SKN-1, whereas the activity of rose essential oil involved SKN-1 but not DAF-16 and the activities of fisetin and phlorizin were mediated by both DAF-16 and SKN-1 [9,10,42,43]. Thus, the intracellular mediators involved in the effects of flavonoids on AD may be compound-specific. In the DM model in this study, butein partially counteracted HGD-induced mortality, and the effect was abolished when expression of *daf-16* and *skn-1* was repressed. Genetic screening has revealed that the decreased survival under HGD was mediated by stress-responsive transcription factors, including DAF-16, DKN-1, and HSF-1, while the preventive effects of fisetin and phlorizin required DAF-16 and SKN-1 [14,15]. Our results showed that butein inhibited dopaminergic neurodegeneration and the accumulation of α-synuclein in a disease model of PD. Further studies focusing on the effects of butein on aging and age-related diseases in higher organisms will lead to the development of clinical applications of butein.

The basic structure of butein is comprised two aromatic rings connected by a group of α, β-unsaturated carbonyl (chalcone) and two hydroxy substituents on each ring. X-Ray structure analysis and quantitative structure activity relationship (QSAR) approaches suggest that the hydroxy groups at the ortho position of the aromatic rings of butein can explain its strong antioxidant activity [44]. QSAR is a computer-aided analytical approach for predicting biological targets and the mode of interactions of molecules based on molecular structure. QSAR analyses for antitubercular, antiviral, and antimalarial activities have been reported with chalcone derivatives [45,46,47]. Because understanding the behavior and molecular targets of molecules is crucial for developing novel therapeutic compounds, detailed QSAR analysis for the anti-aging activity of butein is necessary in the near future.

## 5. Conclusions

Supplementation with butein shows increased survival under oxidative stress, but not under heat stress or UV irradiation. Butein confers a longevity phenotype that is accompanied by reduced fertility and requires BEC-1 and DAF-16. Nuclear localization of DAF-16 and expression of its downstream targets *hsp-16.2* and *sod-3* was observed in long-lived animals treated with butein. Aβ-induced toxicity was reduced by butein, which was dependent on SKN-1. Reduced survival due to HGD was restored by butein, which required DAF-16 and SKN-1. Butein inhibited dopaminergic neurodegeneration and α-synuclein accumulation in a PD model.

## Data Availability

Data are contained within the article.

## Supplementary Materials

The following supporting information can be downloaded at: https://www.mdpi.com/article/10.3390/antiox13020155/s1, Table S1: Effect of butein on lifespan in *C. elegans*; Table S2: Effect of *daf-16* or *bec-1* knockdown on lifespan extension by butein.

## References

[1] Kudryavtseva A.V., Krasnov G.S., Dmitriev A.A., Alekseev B.Y., Kardymon O.L., Sadritdinova A.F., Fedorova M.S., Pokrovsky A.V., Melnikova N.V., Kaprin A.D. (2016). Mitochondrial Dysfunction and Oxidative Stress in Aging and Cancer. Oncotarget.

[2] Finkel T. (2011). Signal Transduction by Reactive Oxygen Species. J. Cell Biol..

[3] Harman D. (1956). Aging: A Theory Based on Free Radical and Radiation Chemistry. J. Gerontol..

[4] Boengler K., Kosiol M., Mayr M., Schulz R., Rohrbach S. (2017). Mitochondria and Ageing: Role in Heart, Skeletal Muscle and Adipose Tissue. J. Cachexia Sarcopenia Muscle.

[5] Aggarwal V., Tuli H.S., Varol A., Thakral F., Yerer M.B., Sak K., Varol M., Jain A., Khan M.A., Sethi G. (2019). Role of Reactive Oxygen Species in Cancer Progression: Molecular Mechanisms and Recent Advancements. Biomolecules.

[6] Shin D.H., Dier U., Melendez J.A., Hempel N. (2015). Regulation of MMP-1 Expression in Response to Hypoxia Is Dependent on the Intracellular Redox Status of Metastatic Bladder Cancer Cells. Biochim. Biophys. Acta.

[7] Mendez E.F., Sattler R. (2015). Biomarker Development for C9orf72 Repeat Expansion in ALS. Brain Res..

[8] Singh A., Kukreti R., Saso L., Kukreti S. (2019). Oxidative Stress: A Key Modulator in Neurodegenerative Diseases. Molecules.

[9] Park S., Kim B.K., Park S.K. (2022). Effects of Fisetin, a Plant-Derived Flavonoid, on Response to Oxidative Stress, Aging, and Age-Related Diseases in Caenorhabditis elegans. Pharmaceuticals.

[10] Park S., Park S.K. (2022). Anti-Oxidant and Anti-Aging Effects of Phlorizin Are Mediated by DAF-16-Induced Stress Response and Autophagy in Caenorhabditis elegans. Antioxidants.

[11] Zhu Q., Qu Y., Zhou X.G., Chen J.N., Luo H.R., Wu G.S. (2020). A Dihydroflavonoid Naringin Extends the Lifespan of C. elegans and Delays the Progression of Aging-Related Diseases in PD/AD Models via DAF-16. Oxid. Med. Cell. Longev..

[12] Okoro N.O., Odiba A.S., Osadebe P.O., Omeje E.O., Liao G., Fang W., Jin C., Wang B. (2021). Bioactive Phytochemicals with Anti-Aging and Lifespan Extending Potentials in Caenorhabditis elegans. Molecules.

[13] Zhou D.D., Luo M., Huang S.U., Saimaiti A., Shang A., Gan R.U., Li H.B. (2021). Effects and Mechanisms of Resveratrol on Aging and Age-Related Diseases. Oxid. Med. Cell. Longev..

[14] Büchter C., Ackermann D., Havermann S., Honnen S., Chovolou Y., Fritz G., Kampkötter A., Wätjen W. (2013). Myricetin-Mediated Lifespan Extension in Caenorhabditis elegans Is Modulated by DAF-16. Int. J. Mol. Sci..

[15] Kumar J., Park K.C., Awasthi A., Prasad B. (2015). Silymarin Extends Lifespan and Reduces Proteotoxicity in C. elegans Alzheimer’s Model. CNS Neurol. Disord. Drug Targets.

[16] Jung C.H., Jun C.Y., Lee S., Park C.H., Cho K., Ko S.G. (2006). Rhus verniciflua Stokes Extract: Radical Scavenging Activities and Protective Effects on H_2_O_2_-Induced Cytotoxicity in Macrophage RAW 264.7 Cell Lines. Biol. Pharm. Bull..

[17] Jung C.H., Kim J.H., Hong M.H., Seog H.M., Oh S.H., Lee P.J., Kim G.J., Kim H.M., Um J.Y., Ko S.G. (2007). Phenolic-Rich Fraction from Rhus verniciflua Stokes (RVS) Suppress Inflammatory Response via NF-kappaB and JNK Pathway in Lipopolysaccharide-Induced RAW 264.7 Macrophages. J. Ethnopharmacol..

[18] Szuster-Ciesielska A., Mizerska-Dudka M., Daniluk J., Kandefer-Szerszeń M. (2013). Butein Inhibits Ethanol-Induced Activation of Liver Stellate Sells through TGF-beta, NFkappaB, p38, and JNK Signaling Pathways and Inhibition of Oxidative Stress. J. Gastroenterol..

[19] Gay N.H., Suwanjang W., Ruankham W., Songtawee N., Wongchitrat P., Prachayasittikul V., Prachayasittikul S., Phopin K. (2020). Butein, Isoliquiritigenin, and Scopoletin Attenuate Neurodegeneration via Antioxidant Enzymes and SIRT1/ADAM10 Signaling Pathway. RSC Adv..

[20] Farias-Pereira R., Zhang Z., Park C.S., Kim D., Kim K.H., Park Y. (2020). Butein Inhibits Lipogenesis in Caenorhabditis elegans. Biofactors.

[21] Ansari M.Y., Ahmad N., Haqqi T.M. (2018). Butein Activates Autophagy Through AMPK/TSC2/ULK1/mTOR Pathway to Inhibit IL-6 Expression in IL-1β Stimulated Human Chondrocytes. Cell. Physiol. Biochem..

[22] Chen Y.H., Yeh C.W., Lo H.C., Su S.L., Hseu Y.C., Hsu L.S. (2012). Generation of Reactive Oxygen Species Mediates Butein-Induced Apoptosis in Neuroblastoma Cells. Oncol. Rep..

[23] Mendonca P., Horton A., Bauer D., Messeha S., Soliman K.F.A. (2019). The Inhibitory Effects of Butein on Cell Proliferation and TNF-alpha-Induced CCL2 Release in Racially Different Triple Negative Breast Cancer Cells. PLoS ONE.

[24] Alshammari G.M., Balakrishnan A., Chinnasamy T. (2018). Butein Protects the Nonalcoholic Fatty Liver through Mitochondrial Reactive Oxygen Species Attenuation in Rats. Biofactors.

[25] Liu P., Pan Q. (2022). Butein Inhibits Oxidative Stress Injury in Rats with Chronic Heart Failure via ERK/Nrf2 Signaling. Cardiovasc. Ther..

[26] Peto R., Peto J. (1972). Asymptotically Efficient Rank Invariant Test Procedures. J. R. Statist. Soc. A.

[27] Kamath R.S., Fraser A.G., Dong Y., Poulin G., Durbin R., Gotta M., Kanapin A., Le Bot N., Moreno S., Sohrmann M. (2003). Systematic Functional Analysis of the Caenorhabditis elegans Genome Using RNAi. Nature.

[28] Gruber J., Tang S.Y., Halliwell B. (2007). Evidence for a Trade-Off between Survival and Fitness Caused by Resveratrol Treatment of Caenorhabditis elegans. Ann. N. Y. Acad. Sci..

[29] Hughes S.E., Evason K., Xiong C., Kornfeld K. (2007). Genetic and Pharmacological Factors that Influence Reproductive Aging in Nematodes. PLoS Genet..

[30] Schlotterer A., Kukudov G., Bozorgmehr F., Hutter H., Du X., Oikonomou D., Ibrahim Y., Pfisterer F., Rabbani N., Thornalley P. (2009). Elegans as Model for the Study of High Glucose- Mediated Life Span Reduction. Diabetes.

[31] Pietsch K., Saul N., Menzel R., Stürzenbaum S.R., Steinberg C.E.W. (2009). Quercetin Mediated Lifespan Extension in Caenorhabditis elegans Is Modulated by age-1, daf-2, sek-1 and unc-43. Biogerontology.

[32] Kampkotter A., Nkwonkam C.G., Zurawski R.F., Timpel C., Chovolou Y., Wätjen W., Kahl R. (2007). Effects of the Flavonoids Kaempferol and Fisetin on Thermotolerance, Oxidative Stress and FoxO Transcription Factor DAF-16 in the Model Organism Caenorhabditis elegans. Arch. Toxicol..

[33] Gems D. (2022). The Hyperfunction Theory: An Emerging Paradigm for the Biology of Aging. Ageing Res. Rev..

[34] Fulle S., Protasi F., Tano G.D., Pietrangelo T., Beltramin A., Boncompagni S., Vecchiet L., Fanò G. (2004). The Contribution of Reactive Oxygen Species to Sarcopenia and Muscle Ageing. Exp. Gerontol..

[35] Toth M.L., Sigmond T., Borsos E., Barna J., Erdélyi P., Takács-Vellai K., Orosz L., Kovács A.L., Csikós G., Sass M. (2008). Longevity Pathways Converge on Autophagy Genes to Regulate Life Span in Caenorhabditis elegans. Autophagy.

[36] Morris B.J., Willcox D.C., Donlon T.A., Willcox B.J. (2015). FOXO3: A Major Gene for Human Longevity—A Mini-Review. Gerontology.

[37] Burnaevskiy N., Sands B., Yun S., Tedesco P.M., Johnson T.E., Kaeberlein M., Brent R., Mendenhall A. (2019). Chaperone Biomarkers of Lifespan and Penetrance Track the Dosages of Many Other Proteins. Nat. Commun..

[38] Sanchez-Blanco A., Kim S.K. (2011). Variable Pathogenicity Determines Individual Lifespan in Caenorhabditis elegans. PLoS Genet..

[39] Caruana M., Cauchi R., Vassallo N. (2016). Putative Role of Red Wine Polyphenols against Brain Pathology in Alzheimer’s and Parkinson’s Disease. Front. Nutr..

[40] Akaishi T., Morimoto T., Shibao M., Watanabe S., Sakai-Kato K., Utsunomiya-Tate N., Abe K. (2008). Structural Requirements for the Flavonoid Fisetin in Inhibiting Fibril Formation of Amyloid Beta Protein. Neurosci. Lett..

[41] Dash R., Emran T.B., Uddin M.M.N., Islam A., Junaid M. (2014). Molecular Docking of Fisetin with AD Associated AChE, ABAD and BACE1 Proteins. Bioinformation.

[42] Yang J., Huang X.B., Wan Q.L., Ding A.J., Yang Z.L., Qiu M.H., Sun H.Y., Qi S.H., Luo H.R. (2017). Otophylloside B Protects against Abeta Toxicity in Caenorhabditis elegans Models of Alzheimer’s Disease. Nat. Prod. Bioprospect..

[43] Zhu S., Li H., Dong J., Yang W., Liu T., Wang Y., Wang X., Wang M., Zhi D. (2017). Rose Essential Oil Delayed Alzheimer’s Disease-Like Symptoms by SKN-1 Pathway in C. elegans. J. Agric. Food Chem..

[44] Okoye I., Yu S., Caruso F., Rossi M. (2021). X-ray Structure Determination, Antioxidant Voltammetry Studies of Butein and 2′,4′-Dihydroxy-3,4-dimethoxychalcone. Computational Studies of 4 Structurally Related 2′,4′-diOH Chalcones to Examine Their Antimalarial Activity by Binding to Falcipain-2. Molecules.

[45] Gomes M.N., Braga R.C., Grzelak E.M., Neves B.J., Muratov E., Ma R., Klein L.L., Cho S., Oliveira G.R., Franzblau S.G. (2017). QSAR-Driven Design, Synthesis and Discovery of Potent Chalcone Derivatives with Antitubercular Activity. Eur. J. Med. Chem..

[46] Chen Z., Li P., Hu D., Dong L., Pan J., Luo L., Zhang W., Xue W., Jin L., Song B. (2019). Synthesis, Antiviral Activity, and 3D-QSAR Study of Novel Chalcone Derivatives Containing Malonate and Pyridine Moieties. Arab. J. Chem..

[47] Xue C.X., Cui S.Y., Liu M.C., Hu Z.D., Fan B.T. (2004). 3D QSAR Studies on Antimalarial Alkoxylated and Hydroxylated Chalcones by CoMFA and CoMSIA. Eur. J. Med. Chem..

