# Supplementary material for: Butein Increases Resistance to Oxidative Stress and Lifespan with Positive Effects on the Risk of Age-Related Diseases in Caenorhabditis elegans
Source: Antioxidants (Basel). 2024 Jan 26;13(2):155. doi: 10.3390/antiox13020155 (PMC10886231; doi:10.3390/antiox13020155)
Supplement: Supplementary file 1 [file antioxidants-13-00155-s001.zip › antioxidants-2827535-supplementary.pdf]

Table S1: Effect of butein on lifespan in *C. elegans*

	**Butein (mM)**	**Mean lifespan (d)**	***P* value[1)]**	**% effect[2)]**
1st experiment	0	22.7		
	1	25.0	< 0.001	10.1
2nd experiment	0	20.7		
	1	23.1	0.002	11.6
3rd experiment	0	20.0		
	1	21.3	0.042	6.5

[1)] *P* value was calculated using the log-rank test by comparing the survival of the untreated control group (0 mM butein) to that of butein-treated group (1 mM butein).

[2)] % effects were calculated by (*C*-*B*)/*C**100, where *B* is the mean lifespan of butein-treated group and *C* is the mean lifespan of the untreated control group.

Table S2: Effect of *daf-16* or *bec-1* knockdown on lifespan extension by butein

		**Mean lifespan (d)**		
	**RNAi**	**Control**	**Butein (1 mM)**	***P* value[1)]**
1st experiment	*daf-16*	15.9	15.2	0.399
	bec-1	24.8	26.7	0.260
2nd experiment	*daf-16*	14.7	14.4	0.255
	bec-1	20.3	21.3	0.911
3rd experiment	*daf-16*	13.9	13.9	0.898
	bec-1	24.2	24.6	0.815

[1)] *P* value was calculated using the log-rank test by comparing the survival of the untreated control group (0 mM butein) to that of butein-treated group (1 mM butein).
